# CMOS-Compatible and Low-Cost Thin Film MACE Approach for Light-Emitting Si NWs Fabrication

**DOI:** 10.3390/nano10050966

**Published:** 2020-05-18

**Authors:** Antonio Alessio Leonardi, Maria José Lo Faro, Alessia Irrera

**Affiliations:** 1 Consiglio Nazionale delle Ricerche—Instituto Processi Chimico-Fisici (CNR-IPCF), Viale F. Stagno D’Alcontres 37, 98158 Messina, Italy; antonio.leonardi@dfa.unict.it (A.A.L.); mariajose.lofaro@dfa.unict.it (M.J.L.F.); 2 Dipartimento di Fisica ed Astronomia, Università di Catania, Via Santa Sofia 64, 95123 Catania, Italy; 3 Consiglio Nazionale delle Ricerche—Istituto per la Microelettronica e Microsistemi (CNR-IMM) UoS Catania, Via Santa Sofia 64, 95123 Catania, Italy

**Keywords:** silicon nanowires, MACE (metal-assisted chemical etching), photoluminescence, photonics

## Abstract

Silicon nanowires (Si NWs) are emerging as an innovative building block in several fields, such as microelectronics, energetics, photonics, and sensing. The interest in Si NWs is related to the high surface to volume ratio and the simpler coupling with the industrial flat architecture. In particular, Si NWs emerge as a very promising material to couple the light to silicon. However, with the standard synthesis methods, the realization of quantum-confined Si NWs is very complex and often requires expensive equipment. Metal-Assisted Chemical Etching (MACE) is gaining more and more attention as a novel approach able to guarantee high-quality Si NWs and high density with a cost-effective approach. Our group has recently modified the traditional MACE approach through the use of thin metal films, obtaining a strong control on the optical and structural properties of the Si NWs as a function of the etching process. This method is Complementary Metal-Oxide-Semiconductors (CMOS)-technology compatible, low-cost, and permits us to obtain a high density, and room temperature light-emitting Si NWs due to the quantum confinement effect. A strong control on the Si NWs characteristics may pave the way to a real industrial transfer of this fabrication methodology for both microelectronics and optoelectronics applications.

## 1. Introduction

Silicon nanowires are emerging in several fields as an innovative building block to overcome different challenges from microelectronics [1,2,3] to energetics [4,5,6], photonics [7,8,9], and sensing [10,11,12]. Starting from microelectronics, a lot of effort has been spent in recent years to find novel solutions that permit to surpass the limitations of the modern-day Moore’s law [13]. In particular, approaching the nanoscale dimension, a natural field of research is the application of silicon nanowires (Si NWs) as a channel for a Field Effect Transistor [14,15,16]. 

Photonics is still considered an opportunity-rich and not totally explored field for the integration of light and silicon, which are the two technologies that changed our century the most. In particular, in order to surpass the limit of the bulk silicon as an indirect band gap material, strategies of quantum confinement have been investigated in the last 20 years. Different approaches were used to address this crucial point with the realization as the use of other integrated material [17], several oxidation and etching processes [18] as well as light-emitting porous silicon [19,20,21] and nanocrystals [22,23]. Despite the extraordinary amount of effort spent in this field, for most of the strategy proposed an industrial realization remain still lacking or very complex. 

Si NWs emerge as a very promising material able to successfully couple the light to an industrially compatible silicon nanomaterial. However, with the standard synthesis methods, such as Vapor–Liquid–Solid (VLS) and Reactive Ion Etching (RIE), the realization of quantum-confined Si NWs is very complex and requires expensive equipment [18]. 

Vapor–Liquid–Solid (VLS) still remains the most diffused Si NWs synthesis strategy. Despite the strong diffusion, this approach suffers from several drawbacks that strongly limit the application of the synthesized Si NWs. The most used catalyst is the gold due to the high crystalline quality of the realized Si NWs and the simple thermodynamics physics of the Si/Au alloy [24,25]. However, the high thermal budget required by this method determines several problems as a gold diffusion inside the Si NWs [26]. The gold impurities act as trap levels, strongly increasing the Shockley–Read–Hall recombination and affecting the performance of Si NWs in electrical, as well as optical, applications [25,27]. The doping is commonly realized during the growth, but, due to the high temperature, a non-uniform radial doping profile is obtained [28]. The diameter dimensions are commonly limited to a few tens of nanometers [29] and, even if reaching the quantum-confined dimension is possible, it leads to a lack of the orientation control on the Si NWs growth [14,30]. Several other catalysts were studied in literature but without solving the main issues of this approach and, in most of the cases, with a final lower quality with respect to the one achieved by using the gold.

Several top-down were analyzed by the scientific community for Si NWs realization. Reactive Ion Etching (RIE) and, in particular, Deep RIE (DRIE) are commonly the most used etching approaches in order to obtain high aspect ratio structures. However, the high cost, the need for expensive dedicated equipment and the limit on 50:1 [31,32] of the aspect ratio determines the difficult to achieve quantum-confined and high-density Si NWs array. Moreover, this approach needs to be coupled with a high-throughput and expensive lithographic process (commonly by electron beam). 

Metal-Assisted Chemical Etching (MACE) is gaining more and more attention as a novel approach able to guarantee high Si NWs quality with a high density and a very cost-effective approach. This method is a wet etching anisotropic approach catalyzed by the use of a high electronegative metal. MACE was first proposed and demonstrated in 2000 by Li and Bohn as a high anisotropic etching approach to obtain porous silicon [33]. In the first method, an Al-covered Si wafer is etched by a solution of HNO_3_:HF as oxidant and etchant, respectively. Some years later, Peng et al. [34] demonstrated the possibility to realize a high density of vertically aligned Si NWs with this approach by using an AgNO_3_:HF aqueous mixture. This method is known as the silver salts approach and takes advantage of the silver nanoparticle random precipitation onto the Si surface to catalyze the etching. The average diameter of the realized NWs is usually of about 70 ± 20 nm [35,36] by using the silver salt approach. This method is fast, does not need complicated sample preparation and it is very cheap compared to lithography approaches. With respect to VLS, it is simpler to obtain a high density of Si NWs and it does not require complicated equipment and a high temperature. The MACE approach arises as the most cost-effective approach, as highlighted by several groups [37,38].

However, by using silver salts, the Si NWs diameter is not suitable for the quantum confinement effect and is limited to several tens (>50 nm commonly) of nanometers. Furthermore, during the process, the presence of Ag dendrites is attested onto the samples. The subsequent etching of these dendrites affects and damages the Si NWs. 

In order to surpass the diameter limit of the Si NWs obtained by silver salt MACE, our group has already engineered an innovative metal thin film approach. In particular, Au or Ag layers of a few nanometers, were successfully employed to obtain high crystalline quality and room temperature, light-emitting Si NWs that have been used for several applications. 

In this paper, the thin film MACE approach will be studied in detail, analyzing all the most important approach characteristics of the obtainable Si NWs. We will present the main structural and optical characteristics as a function of the doping, the Si crystalline orientation, and the length. The high flexibility of the approach with the possibility to obtain perpendicular and even tilted Si NWs growth with respect to the substrate will be presented, as well as how to improve the photoluminescence (PL) emission by increasing the length of the Si NWs. A study on the structural and optical characteristics is of key interest to demonstrate the real advantages of this novel fabrication strategy. Indeed, this approach is very cost effective and compatible with the current Complementary Metal-Oxide-Semiconductors (CMOS) technology. A strong control on the Si NWs structural and optical characteristics may pave the way to a real industrial transfer of this fabrication methodology for both microelectronics and optoelectronics applications.

## 2. Materials and Methods

### 2.1. Materials and Chemicals

Standard 4” commercial Si wafer (100) and (111)-orientation and with *n*-type or *p*-type doping (resistivity ~ 1–5 Ω·cm) and 500 μm of thickness were purchased from Siegert Wafer (Aachen, Germany). Hydrofluoric acid (HF) 40% was obtained by Honeywell (Charlotte, NC, USA), while hydrogen peroxide (H_2_O_2_) was obtained from by Sigma-Aldrich (Darmstadt, Germany). The watery solutions were prepared by using by Milli-Q Deionized water (resistivity ~ 18 MΩ·cm). 

### 2.2. Silicon Nanowires Fabrication

Si NWs were fabricated through the use of the thin film Metal-Assisted Chemical Etching (MACE). First of all, native silicon oxide was etched by a watery solution of 2.5M of HF. Subsequently, a thin metal film of 2 nm of Au or 10 nm of Ag was deposited onto the cleaned substrates by an Electron Beam Evaporator (EBE, modified-KS1000SA, Kenosistec, Binasco, Italy) in a high vacuum condition (<10^−6^ mbar) and at room temperature. The samples were then immersed in an aqueous solution of HF (5M) and H_2_O_2_ (0.44M). The metal (Au or Ag) is more electronegative than Si and extract electrons (or inject holes) from the Si substrate that is oxidized by the hydrogen peroxide. The hydrofluoric acid selectively etch the SiO_2_ formed under the metal. Anisostropic etching happens under the metal mask and the Si NWs are formed in the unetched silicon regions. Finally, the metal can be simply removed by a gold etchant (KI solution) or a HNO_3_ solution for Au or Ag, respectively. All the etching is performed at room temperature and previous studies demonstrate the absence of Au or Ag contamination inside the silicon [39]. 

### 2.3. Structural and Optical Analysis Methods

Structural analysis of the fabricated Si NWs was performed by a field-emission Scanning Electron Microscope (SEM, Gemini Supra 25, Zeiss, Oberkochen, Germany). The photoluminescence (PL) spectra were obtained by exciting the NWs with the 364 nm line of an Ar+ laser through a 100x (NA = 0.9) objective and a power of about 100 µW onto the sample plane if it is not differently specified. The signal is collected in back scattering configuration through the same objective, spectrally decomposed by a 600 L/mm grating, and acquired by a Peltier −70°C cooled CCD (Synapse). 

### 2.4. Data Treatment

All the linear best fits reported here show a Pearson coefficient higher than 0.98, demonstrating good linear trend behavior. The length measurement for the etching rate as well as the optical properties in terms of Photoluminescence (PL) emission were obtained as the average value on several (3 at least) NWs samples and for each of them in different points of the sample. This statistic permitted to obtain the reported error bars on the measurements. Both the linear fit and the Gaussian one are obtained through the Origin software libraries. 

The PL signals as a function of the power are shown for simplicity in power density considering the measured laser spot. The laser pump fluency reported (photon flux for area and time unit, used in Figure 4d) takes into account a continue laser source and the laser wavelength of 364 nm with the following equation:

(1) $$\Phi =\frac{Power\;}{Area\;}\frac{\lambda }{hc}\frac{1}{second}$$

where *λ* is the wavelength, *h* the Planck constant, and *c* the speed of light velocity.

## 3. Results and Discussion

**Figure 1:**
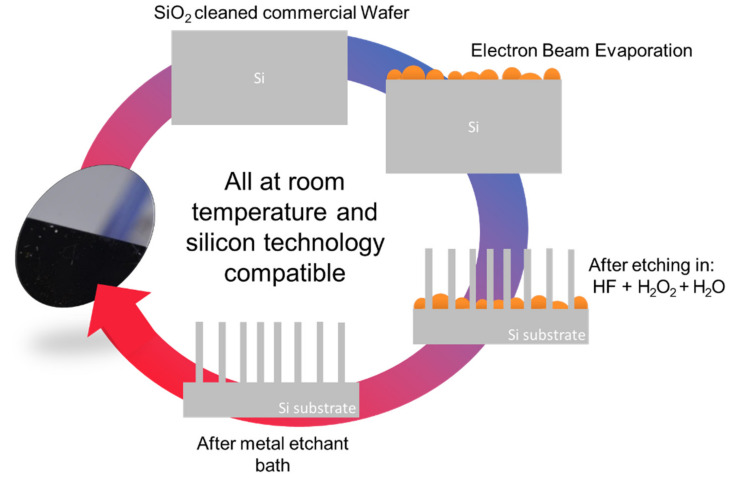
Scheme of the thin metal film Metal-Assisted Chemical Etching for the realization of silicon nanowires (Si NWs).

As shown in Figure 1, a discontinuous thin metal film is deposited by Electron Beam Evaporation (EBE) on an Si substrate. By using room temperature electron beam evaporation, the deposited metal atoms organize themselves in a percolative morphology compared to different techniques, such as sputtering, that have a more rapid deposition rate and coverage.

The sample is then immersed into a watery solution of H_2_O_2_:HF and the metal drive the oxidation just underneath the Si that is subsequently etched by the HF. Silicon nanowires are formed on the uncovered regions and the metal is finally removed by a proper etchant solution. All the processes are at room temperature and the metal does not diffuse inside the silicon. In this approach, the hydrogen peroxide is used as oxidation agent. As for the standard Metal-Assisted Chemical Etching (MACE), we can consider the Si as a local anode with respect to the metal (acting as cathode) for the current produced in the Si/metal interface. Further details on the chemicals used can be found in the Section 2.

By changing the etching time, it is possible to vary the Si NWs length from a few hundred nanometers to several micrometers. Therefore, the density of the Si NWs is huge, about 10^12^ NWs/cm^2^, and this is a crucial point for all the applications. The average diameter of the synthesized Si NWs is determined by the thickness and the type of the metal used as a catalyst. In fact, the thin metal layer is discontinuous and nanometric areas of uncovered silicon are present. The average dimension of these areas is determined by the material wettability and thickness [39,40]. The Au percolative morphology reproducibility was tested performing several deposition and Si NWs fabrication test obtaining the same average morphology and PL emission spectra. All these points demonstrate a good reproducibility of the process.

**Figure 2:**
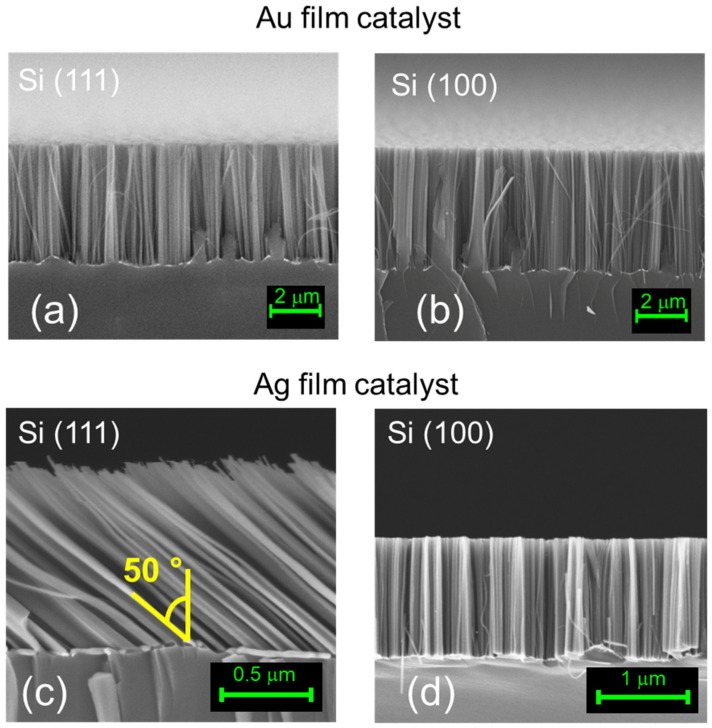
Cross-section Scanning Electron Microscope (SEM) image of vertically aligned Si NWs realized by using a 2 nm Au film on Si (111) (**a**) and Si (100) (**b**) crystalline orientation. (**c**,**d**) are the cross-section SEM images obtained for the case of 10 nm of Ag as catalyst for the case of Si (111) and Si (100), respectively.

**Figure 3:**
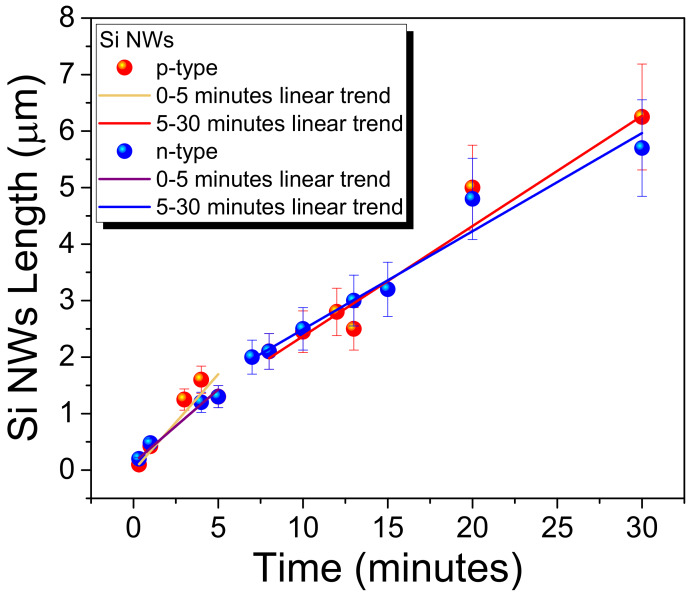
Si NWs length as a function of the etching time for *p*-type substrates (red dots) and *n*-type substrates (blue dots). Both *n*- and *p*-type correspond to a resistivity of about 1–5 Ω·cm.

This method offers great fabrication flexibility. Indeed, as a function of the type of metal used and of the Si substrate crystalline orientation (with the same etching solution), it is possible to obtain different Si NWs orientations. In Figure 2, a cross section (a) and a plan view (b) SEM image of the typical NWs obtained by using 2 nm of gold are reported for the case of (100)-orientation. However, in this case, independent of the type of orientation tested for both (100) and (111), vertically aligned Si NWs are obtained with just a slightly change on the in plane morphology [40]. By using 10 nm of Ag, it is possible to obtain different NWs growth directions as a function of the Si crystalline orientations. Indeed, in Figure 2c,d, the cross-section SEM images for the case of (111) and (100) Si substrate are respectively reported. This orientation change can be ascribed to the difference in the oxidation rate with respect to the etching one. In the Si (111) substrate, more Si atoms are exposed in the surface, and this is well known for the silicon oxidation process strongly used in industry (about 7 × 10^14^ atoms/cm^2^ and 8 × 10^14^ atoms/cm^2^ for (100)- and (111)-oriented substrate, respectively) [27]. The difference in the second case is linked to the electronegativity of the silver with respect to the gold that will determine the oxidation rate together with the oxidation agent concentration (in this case H_2_O_2_ concentration that is fixed). Indeed, for the same doped substrate, the final etching rate without changing the HF concentration is strongly dependent on the oxidation efficiency of the metal. This effect is well known in the literature of the standard MACE process, where it can be obtained and controlled by changing the oxidant agent concentration [41]. Our results are in agreement with the literature, and, as commonly happens without a very high H_2_O_2_ concentration for the (111)-oriented substrates, the etch proceed along the (100) direction with a 50° angle with respect to the substrate (as visible in Figure 3) [41,42].

Defining the etching rate, *R_etch_*, as the number of atoms of Si etched by each atom of metal per second follows:

(2) $$R_{etch}=\frac{velocity_{etch}}{thickness_{metal}}\frac{\rho_{Si}}{\rho_{metal}}$$

where the thickness of the metal is the nominal thickness of the deposited catalyst and the *ρ_Si_* and *ρ_metal_* represent the density of silicon and of the metal (Au or Ag), respectively.

From this equation, considering an average etching velocity of about 7.7 nm/s (as will be shown in Figure 3) for the (100) substrate and the use of 2 nm of Au or 10 nm of Ag, an etching rate of 0.5 atoms/s for the case of the gold and about 0.2 atoms/s for the case of Ag can be found. Au is 2.5 times more efficient than Ag. As elicited, this difference can be ascribed to the different electronegativity and oxidation number of Au and Ag. 

Since the thin film MACE Si NWs are very thin and have a very high aspect ratio, for several micrometers in length (commonly > 6 µm), the formation of bundles of NWs tips can occur. This can be attributed to the surface tension forces exerted on the NWs tips during the drying of the sample, which is a common phenomenon for drying of long nanowire arrays from the solvent. According to several groups, this phenomenon can be avoided with an isopropanol bath and gentle drying or with the use of super-critical CO_2_ drying [43]. In particular, the use of super-critical drying seems to be the best strategy to obtain the highest quality, as demonstrated by the industrial use of this approach for the realization of MEMS in the CMOS technology [44]. However, up to 3 µm of length in Si NWs, we did not observe this phenomenon and no tip bundle were observed.

As elicited, the doping type and level in these types of fabrication processes may affect the etching rate and therefore the final NWs length as a function of the etching time. In Figure 3, the length of the obtained Si NWs for the (100)-orientation, as well as the case of n- and p-doping with a resistivity around 1–5 Ω·cm in both cases (doping concentration around 10^15^–10^16^ atoms/cm^3^), is reported as a function of the etching time. 

As is visible in Figure 3, the etching seems to be characterized by two different regimes, a first one for time under about 5 min and a second one for longer times. Indeed, trying to fit the trend of both the two types of doping, two slightly different linear regimes can be found. In the first one, we have a higher angular coefficient that is about 0.34 ± 0.05 µm/min for *p*-type and 0.26 ± 0.03 µm/min for *n*-type. These angular coefficients go down to 0.20 ± 0.03 µm/min for *p*-type and 0.17 ± 0.01 µm/min for *n*-type for longer etching times. For these lightly doped substrates, just a slightly difference in the etching time can be observed in favor of a faster *p*-type etching. However, as shown by the Figure 3 the etching time difference is so small that for practical processes can be considered negligible. These two regimes behavior of the etching is in perfect agreement with the standard MACE literature [45].

One of the most interesting point of this fabrication method is the possibility to obtain quantum-confined Si NWs that exhibit photoluminescence (PL) at room temperature (RT).

**Figure 4:**
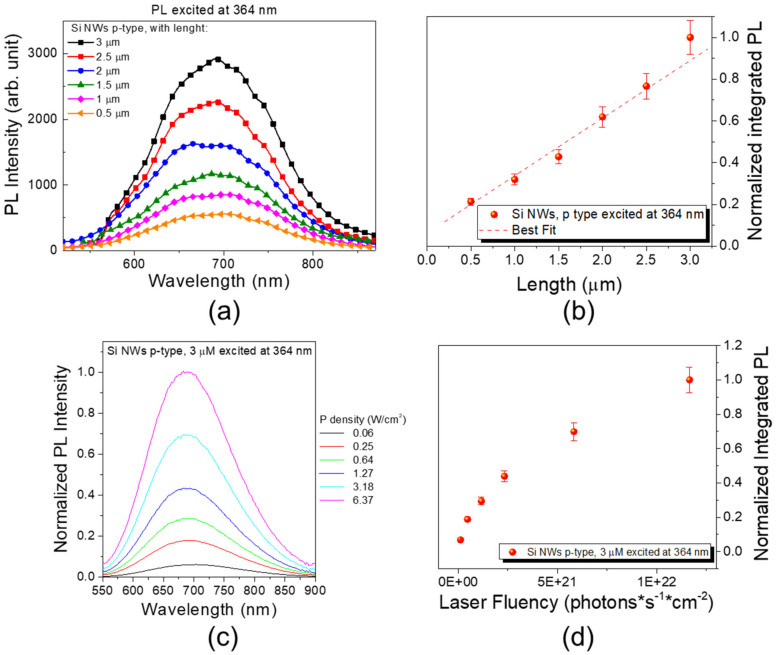
All the photoluminescence (PL) measurement were obtained through a 364 nm excitation. (**a**) Si NWs PL emission as function of the length for *p*-type substrates. (**b**) Integrated PL signal normalized to the highest one (3 µm long Si NWs) as a function of the NWs length. (**c**) PL signal obtained from the Si NWs sample as a function of the power density (or laser fluency). The signals are normalized to the highest one obtained with the highest power density. (**d**) Integrated PL signals normalized to the highest excitation fluency are reported as a function of the laser fluency.

Indeed, the RT PL emission is attested by the spectra in Figure 4 and is so bright that it can be observed by naked eye. In the past, we have already shown that the emission of the Si NWs is due to quantum confinement effect [46] and some application of their bright emission [10,11,47].

Once we had characterized the key fabrication parameters of this method, we spent our efforts on the study of their influence on the optical characterization. In particular, we have studied the PL intensity behavior as a function of NWs length and power density (or photon flux) excitation in continued excitation with a 364 nm line of Ar^+^ laser. In Figure 4a, the spectra of the obtained Si NWs sample from a (100)-oriented p-doped Si substrate are reported as a function of the NWs length. The PL intensity is reported normalized to the brightest one (the signal obtained from the 3 µm long Si NWs). We found that 3 μm is an optimal length that permits one to optimize the Si NWs PL properties without having tips bunching phenomena for the Si NWs that should need complex equipment, such as critical point dryer.

After the etching rate study, we tested the PL response of some of the obtained sample. As is possible to observe, an increasing PL emission follows the NWs length increases. The integrated signal was obtained by a Gaussian fit of the NWs PL emission. In particular, in Figure 4b, the trend of the integrated PL responses is reported normalized to the highest obtained one (3 µm long Si NWs) as a function of the NWs length. In particular, an average increase of a 1.36 factor was observed increasing the length of 500 nm. Indeed, a linear trend can be identified as shown by the red dashed line in the graph. Hence, a linear increasing of the PL is attested increasing the NWs length from few hundreds of nanometers up to 3 µm. In particular, going from 500 nm to 1 µm, a 1.5 factor increasing was found for the PL signal, while this increase become a 1.34 factor between 1.5- and 1-µm-long Si NWs. Increasing the length from 1.5 to 2 µm follows a 1.44 factor increase in the PL. Finally, this PL increase corresponds to a 1.24 and 1.29 factor going from 2 to 2.5 µm and from 2.5 to 3 µm, respectively.

In Figure 4c, the PL signal from the 3 µm long *p*-type Si NWs sample is reported as a function of the excitation power density by using a 364 nm laser. The spectra are reported normalized to the highest one corresponding to the highest power density used (6.37 W/cm^2^), as is it possible to observe the PL emission of the Si NWs increases going from 0.06 to 6.37 W/cm^2^. To study the trend behavior, each luminescence signal was fitted by a Gaussian. In Figure 4d, all the obtained integrated signals were reported normalized to the highest one (6.37 W/cm^2^ = 1.16 × 10^22^ photons · s^−1^ · cm^−2^) and in terms of laser pump fluency.

In particular, increasing the power density from 0.06 to 0.25 W/cm^2^, a 2.71 factor increase was found for the PL signal, while this increase became a 1.53 factor between 0.25 and 0.64 W/cm^2^ power density. Increasing the power density from 0.64 to 1.27 W/cm^2^ follows a 1.52 factor increase in the PL and from 1.27 to 3.18 W/cm^2^ it follows a 1.59 factor gaining. Finally, this PL increase corresponds to a 1.42 factor going from 3.18 to 6.37 W/cm^2^ that indicates a sublinear trend with a saturation behavior typical of optical losses. Indeed, as is possible to observe, a sublinear trend for the PL emission as function of the photon flux is visible. The photon flux corresponding to the used power density are reported in Table 1. The saturation effect observed for high fluencies may be due to the continued laser excitation that induces thermal effects, as increasing phonon scattering affects the carrier lifetime [27]. 

## 4. Conclusions

In this paper, a detailed study of the structural and optical characteristic of ultrathin Si NWs is reported as a function of the etching process parameters. This modified approach surpasses the limit of the traditional MACE method through the use of thin metal films, permitting us to obtain RT, light- emitting Si NWs. This method is CMOS-technology compatible, low-cost, and permits one to obtain a high density of Si NWs with perpendicular or tilted orientation as a function of the substrate crystalline orientation and the metal used in fixed solution concentration. Indeed, for the case of (111)-oriented substrate, the obtained Si NWs results are 50 degree tilted with respect to the substrate plane by changing the catalyst from the 2 nm of gold to 10 nm of silver. The etching rate was studied by changing the doping founding a two regimes behavior with a faster etching rate in the first 5 min and a slightly slower one in the second phase. Moreover, this etching rate was to be found very similar for lightly p- or n-doped substrate (10^15^–10^16^ dopant atoms/cm^3^). As reported by the optical measurement, the PL was found to linearly increase with a factor of about 1.36 for each 500 nm length increase. Moreover, the PL response of this sample was studied as a function of the laser power fluency funding the typical sublinear semiconductor behavior in perfect agreement with the literature. In conclusion, the high flexibility of this approach and the strong control on the Si NWs characteristics, coupled with its low cost and compatibility with the current Si industry approaches, may pave the way to a real industrial transfer of this fabrication methodology for both microelectronics and optoelectronics applications.

## Figures and Tables

**Table 1:** Power density to laser photon flux conversion table.

Power Density (W/cm^2^)	Laser Fluency (Photons · s^−1^ · cm^−2^)
0.06	1.16 × 10^20^
0.25	4.66 × 10^20^
0.64	1.16 × 10^21^
1.27	2.33 × 10^21^
3.18	5.82 × 10^21^
6.37	1.16 × 10^22^
